# Prevalence of peripheral neuropathy defined by monofilament insensitivity in middle-aged and older adults in two US cohorts

**DOI:** 10.1038/s41598-021-98565-w

**Published:** 2021-09-27

**Authors:** Caitlin W. Hicks, Dan Wang, B. Gwen Windham, Kunihiro Matsushita, Elizabeth Selvin

**Affiliations:** 1 Division of Vascular Surgery and Endovascular Therapy, Johns Hopkins University School of Medicine, Baltimore, MD USA; 2 Department of Epidemiology, Johns Hopkins Bloomberg School of Public Health, 2024 E. Monument St., Suite 2-600, Baltimore, MD 21287 USA; 3 Department of Medicine, University of Mississippi Medical Center, Jackson, MS USA

**Keywords:** Medical research, Neurology, Risk factors

## Abstract

Peripheral neuropathy is associated with substantial morbidity, but risk factors other than diabetes are largely uncharacterized. The aim of this study was to describe the prevalence and risk factors for peripheral neuropathy in adults with and without diabetes from two different population-based studies in the US. We performed a cross-sectional analysis of 5200 black and white participants from NHANES (1999–2004, age 40–85 years) and 3362 black and white participants from the ARIC Study (2016–2017, age 70–89 years) who underwent monofilament testing for peripheral neuropathy using a shared protocol. We used logistic regression to quantify age, sex, and race-adjusted risk factor associations for peripheral neuropathy among middle-aged (40–69 years) and older (≥ 70 years) adults. The age, sex, and race-adjusted prevalence of peripheral neuropathy (decreased sensation on monofilament testing) was 10.4% for middle-aged adults in NHANES, 26.8% for older adults in NHANES, and 39.2% for older adults in ARIC. Diabetes was an important risk factor, but more strongly associated with peripheral neuropathy in middle-aged (OR ~ 5 for long-standing diabetes) compared to older adults (ORs ~ 1.5–2). Male sex (ORs ~ 2), black race (ORs ~ 1.3–1.5), and greater height (ORs ~ 1.5–3) were robust risk factors for peripheral neuropathy. Other risk factors included body mass index, education, and peripheral artery disease. The burden of peripheral neuropathy defined by abnormal monofilament testing among older adults is substantial, even among adults without diabetes. Studies are needed to understand the etiology and prognosis of peripheral neuropathy in the absence of diabetes.

## Introduction

Peripheral neuropathy (PN) resulting in decreased lower extremity sensation is a common sequela of diabetes^1–5^. The consequences of PN can be devastating, including foot ulcers, major amputation, falls, intracranial injuries, and impaired quality of life^6–8^. We have recently shown that PN is independently associated with mortality in the general population, including among adults both with and without diabetes^9^. Despite substantial associated morbidity, the epidemiology and risk factors associated with PN in older adults are relatively uncharacterized, especially in adults without a history of diagnosed diabetes. There have been a number of prior cohort studies describing the epidemiology and risk factors associated with PN^10–17^. However, previous studies have typically focused on middle-aged adults or persons with diabetes.

Monofilament testing is an inexpensive, easy-to-use test to screen for PN. According to the American Diabetes Association, the 10-g monofilament test is the gold standard test to diagnose decreased lower extremity sensation resulting from PN^18^. Prospective studies have shown that decreased sensation to monofilament testing is highly predictive of foot ulceration^19,20^, and thus is an essential component of diabetic foot exams^21^. Age is a risk factor for PN, but age-related increases in the prevalence of PN in persons without diabetes and risk factors for PN in older adults have not been a focus in prior studies.

One of the only large US population-based studies to implement standardized monofilament testing was the 1999–2004 National Health and Nutrition Examination Survey (NHANES)^16,17^. The Atherosclerosis Risk in Communities Study (ARIC) is a community-based study that has collected longitudinal medical, social, and demographic data from more than 15,000 participants over the past 30 years. Between 2016 and 2017 (ARIC visit 6), all ARIC participants underwent standardized monofilament testing for PN. In contrast to NHANES, which enrolled participants ≥ 40 years of age, ARIC participants were ≥ 70 years of age at the time of monofilament testing. As a result, ARIC offers a unique opportunity to study the prevalence and risk factors associated with PN in a sample of older adults. The aim of this study was to describe the prevalence of and risk factors for PN, defined by monofilament insensitivity, in middle-aged and older adults with and without diabetes from NHANES and ARIC.

## Methods

### Study populations

The NHANES are cross-sectional complex samples of the US civilian non-institutionalized population conducted by the National Center for Health Statistics. We examined data from black and white participants from NHANES 1999–2004, which included a standardized monofilament exam in participants aged 40 years and older. Among the 5771 NHANES participants aged ≥ 40 years of age who had monofilament testing data available, we excluded those who were missing any variables of interest (N = 578). NHANES study protocols were approved by the Institutional Review Board of the National Center for Health Statistics. All participants provided written informed consent. All research was performed in accordance with the Declaration of Helsinki.

ARIC is a community-based study of 15,792 adults enrolled between 1987 and 1989 from four US communities (Forsyth County, North Carolina; Jackson, Mississippi; Minneapolis, Minnesota; and Washington County, Maryland) and followed prospectively with serial visits. We used ARIC visit 6 data (collected between 2016 and 2017) for the current study because this visit included a standardized monofilament assessment. Among the 4003 black and white participants who attended ARIC visit 6, we excluded participants who were missing monofilament testing information (N = 386), and those who were missing any other variables of interest (N = 255). The ARIC study was approved by the Institutional Review Boards at each study site and informed consent was obtained from all participants.

### Measurement of peripheral neuropathy using monofilament testing

In both NHANES and ARIC, the lower extremity monofilament exam was performed by trained technicians using a standard monofilament (5.07 Semmes–Weinstein nylon monofilament mounted on a plastic handle, delivering approximately a 10-g filament force) to apply slight pressure to the bottom of each participant’s feet at the following three sites: (1) plantar-first metatarsal head; (2) plantar-fifth metatarsal head; and (3) plantar-hallux. If the participant’s first response at a site was correct, the test was not repeated at that site. If the participant could not correctly identify when the pressure was applied, the test was repeated at that site for up to two times until a total of two similar responses were obtained (up to three tests at each site). A site was defined as insensate if two incorrect or undeterminable responses were given by the participant. PN was defined as having at least one insensate site on the left or right foot, corresponding to a reduced sensation to touch.

Of note, there was a monofilament examination instrument change during the early part of ARIC visit 6 (October 10th, 2016) due to the discontinuation by the manufacture of previous instrument (5.07 AliMed reusable nylon Semmes–Weinstein Monofilament). The effect of this instrument change was evaluated with a cross-over study of 80 participants (20 from each ARIC Field Center). The results showed that there was no significant insensate classification or prevalence of PN differences between the two instruments.

### Other variables

In NHANES, age, sex, race, education, smoking status, and drinking status were self-reported. Diabetes was defined as self-reported doctor-diagnosed diabetes, current glucose-lowering medication use, or HbA1c ≥ 6.5%. Among those without diabetes, prediabetes was defined as HbA1c level between 5.7% and 6.4% and normal was defined as HbA1c < 5.7%. Body mass index (BMI) was calculated as weight (kilograms) divided by squared height (m^2^). Cardiovascular disease was defined as self-reported doctor diagnosed history of coronary heart disease, heart attack, or stroke. Hypertension was defined as systolic blood pressure (SBP) ≥ 140 mmHg, diastolic blood pressure (DBP) ≥ 90 mmHg, or taking blood pressure control medication. Hypercholesterolemia was defined as total cholesterol ≥ 240 mg/dL or taking cholesterol control medication. Peripheral artery disease was defined as left or right ankle brachial index (ABI) < 0.9. Cancer was defined as self-reported doctor diagnosed any cancer or malignancy other than melanoma skin cancer.

In ARIC, sex, race and education were ascertained at visit 1 (1987–1989). Unless otherwise noted, all other data used in the present study were collected at visit 6 (2016–2017). Age, smoking status and drinking status were self-reported. Diabetes was defined as self-reported doctor-diagnosed diabetes, current glucose-lowering medication use, or HbA1c ≥ 6.5% at visit 6. Pre-diabetes was defined as HbA1c level between 5.7% and 6.4% among those without diabetes. BMI was calculated as weight (kilograms) divided by squared height (m^2^). Cardiovascular disease was defined as any occurrence of coronary heart disease, stroke, or heart failure, which included any validated hospitalized myocardial infarction, fatal coronary heart disease, cardiac procedure or electrocardiogram myocardial infarction, hospitalization or death due to heart failure, or definite or probable stroke at or prior to visit 6. Hypertension was defined as SBP ≥ 140 mmHg, DBP ≥ 90 mmHg, or taking blood pressure control medication. Hypercholesterolemia was defined as total cholesterol ≥ 240 mg/dL, or taking cholesterol control medication. Peripheral artery disease was defined as left or right ABI < 0.9. SBP, DBP, total cholesterol and ABI information were collected at visit 6. Cancer was defined as any invasive cancer and cancer deaths, which included adjudicated bladder, breast, colorectal, liver, lung, pancreatic and prostate cancer, and other non-adjudicated cancers, but not melanoma skin cancer, from cancer registry and death certificate at or prior to visit 6.

### Statistical analyses

We examined the characteristics of US adults from NHANES (1999–2004) by two age categories, approximately middle-aged (40–69 years) and older aged (≥ 70 years), and ARIC visit 6 (2016–2017) participants (all of whom were ≥ 70 years of age). The age categories for NHANES were chosen to match the older age range of ARIC participants at visit 6 (i.e. ≥ 70 years of age). We estimated the age-, sex- and race-adjusted prevalence and standard errors of PN overall and by participant characteristics for the above two age groups in NHANES and ARIC visit 6 separately. We used the logistic regression models to evaluate the age-, sex- and race-adjusted associations of various participant characteristics with PN. We also used logistic regression to identify risk factors associated with PN after mutually adjusting for all variables. Of note, the ABI and cancer variables had a high proportion of missing. Thus, analyses of these data were conducted in a smaller number of participants (N = 4419 for peripheral artery disease in NHANES; N = 2971 for peripheral artery disease in ARIC; and N = 3136 for cancer in ARIC). Finally, we used logistic regression to evaluate the age, sex, and race-adjusted continuous association of PN with diabetes duration categories (0–4 years, 5–14 years, 15–24 years, and ≥ 25 years).

We performed all analyses using Stata, version 15.1 (StataCorp) with P < 0.05 denoting statistical significance. For all analyses of NHANES data, we incorporated sampling weights in the Stata survey commands to obtain unbiased estimates from the complex sampling design and used Taylor series (linearization) method to estimate the standard errors, as recommended by the National Center for Health Statistics analysis guidelines. All estimates from NHANES presented in the results and the tables/figures are nationally representative of the US adult population in the age groups examined. Estimates from ARIC are unweighted.

## Results

### Characteristics of study participants

Overall, 5193 middle-aged (40–69 years, N = 3578) and older (≥ 70 years, N = 1615) NHANES participants and 3362 older (≥ 70 years) ARIC participants underwent standardized lower extremity monofilament testing. The ARIC Study oversampled African Americans so more participants were black as a function of the study design (20.4% in ARIC vs. 6.5% US adults in NHANES) and ARIC participants were older (i.e., larger percentage of the ARIC population was ≥ 80 years of age compared to US adults: 42.0% vs. 30.6%) (Table 1). The prevalence of prediabetes was similar in older US adults and in the ARIC Study population, whereas diabetes was more common in ARIC participants ≥ 70 years compared to US adults in this age group (31.8% vs 16.8%).

**Table 1:** Characteristics of US adults aged 40–69 and ≥ 70 years (NHANES, 1999–2004) and ARIC participants aged ≥ 70 years (Visit 6, 2016–2017).

	Aged 40–69 years	Aged 70 years or older	
	US adults (NHANES) (N = 3578)	US adults (NHANES) (N = 1615)	ARIC (Visit 6) (N = 3362)
**Age, years**			
40–49	43.0%	–	–
50–59	34.0%	–	–
60–69	23.0%	–	–
70–74	–	38.3%	14.8%
75–79	–	31.1%	43.2%
≥ 80	–	30.6%	42.0%
Male	49.7%	41.5%	41.1%
Black	10.7%	6.5%	20.4%
**Education**			
Less than high school	13.8%	29.3%	11.1%
High school or vocational school	26.5%	30.9%	41.3%
College and above	59.7%	39.8%	47.6%
**Diabetes status**			
Normal	77.1%	59.0%	43.4%
Pre-diabetes	13.6%	24.2%	24.8%
Diabetes (< 10 years duration)	6.3%	8.9%	23.0%
Diabetes (≥ 10 years duration)	3.1%	7.9%	8.8%
**Body mass index, kg/m**^**2**^			
0–24.9	28.6%	33.6%	28.9%
25–29.9	36.3%	40.8%	38.9%
≥ 30	35.1%	25.6%	32.2%
**Sex-specific height quartile**			
Q1	15.5%	39.5%	23.9%
Q2	23.5%	27.4%	23.0%
Q3	28.0%	20.1%	25.1%
Q4	33.0%	13.0%	28.0%
**Smoking status**			
Never	44.6%	51.0%	45.0%
Former	32.4%	42.2%	48.3%
Current	23.0%	6.8%	6.7%
**Drinking status**			
Never	9.9%	20.6%	20.4%
Former	19.7%	30.7%	28.3%
Current light/moderate drinker	40.2%	40.3%	46.5%
Current heavier drinker	30.2%	8.4%	4.8%
Prevalent cardiovascular disease	8.1%	24.8%	20.0%
Hypertension	37.1%	74.5%	84.0%
Hypercholesterolemia	33.4%	45.2%	60.5%
Prevalent chronic kidney disease	11.4%	50.4%	41.2%
Peripheral artery disease^a^	3.3%	17.0%	5.2%
Cancer^b^	9.0%	25.2%	15.9%†

^a^Percentages are among participants who were not missing data for peripheral artery disease: N = 3215 in NHANES aged 40–69 years, N = 1204 in NHANES aged ≥ 70 years, and N = 2971 in ARIC visit 6.

^b^Percentage for the ARIC study is among participants who were not missing cancer status at ARIC visit 6, N = 3136.

### Prevalence of and risk factors for peripheral neuropathy

**Figure 1:**
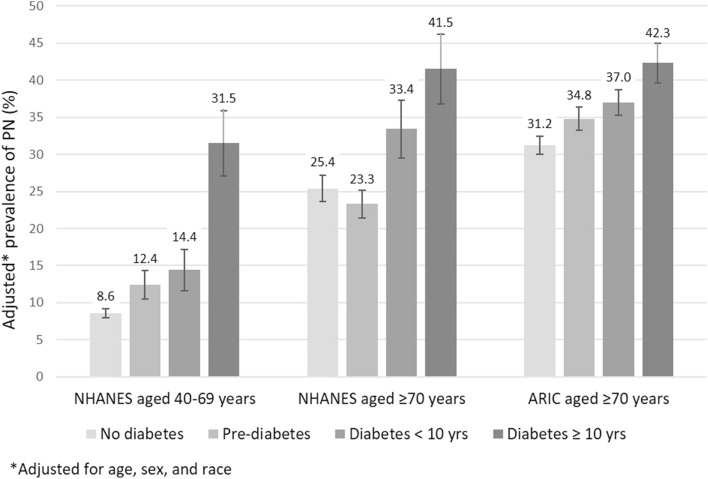
Age, sex, and race-adjusted prevalence of peripheral neuropathy stratified by diabetes status in US adults aged 40–69 and ≥ 70 years (NHANES, 1999–2004) and ARIC participants aged ≥ 70 years (Visit 6, 2016–2017).

The overall prevalence of PN defined by monofilament insensitivity in the general US population aged 40 years or older was 13.5%, equivalent to 18.6 million US adults in 2010. The overall prevalence of PN in adults aged 40 or older was 28.4% in persons with diabetes (4.2 million) and 11.8% in those without diabetes (14.4 million). The age-, sex-, and race-adjusted estimated prevalence of PN in US adults was 10.4 ± 0.5% in those 40–69 years and 26.8 ± 1.1% among those ≥ 70 years. In the ARIC Study, the prevalence of PN was 34.4 ± 0.8%. The prevalence of PN was higher at older ages (Fig. 1), with 34.2 ± 1.8% and 42.4 ± 1.3% of NHANES and ARIC participants ≥ 80 years of age affected, respectively (Table 2).

**Table 2:** Age, sex, and race-adjusted prevalence [% (SE)] of peripheral neuropathy by demographic and risk factor categories among US adults aged 40–69 and ≥ 70 years (NHANES, 1999–2004) and ARIC participants aged ≥ 70 years (Visit 6, 2016–2017).

	Aged 40–69 years	Aged 70 years or older	
	US adults (NHANES) (N = 3578)	US adults (NHANES) (N = 1615)	ARIC (Visit 6) (N = 3362)
Overall	10.4 (0.5)	26.8 (1.1)	34.4 (0.8)
**Age**			
40–49	6.4 (0.6)	–	
50–59	10.9 (0.9)	–	
60–69	17.5 (1.3)	–	
70–74	–	20.7 (1.9)	24.5 (1.9)
75–79	–	27.5 (2.1)	30.1 (1.2)
≥ 80	–	34.2 (1.8)	42.4 (1.3)
**Sex**			
Female	6.7 (0.6)	20.5 (1.4)	25.3 (1.0)
Male	14.2 (0.7)	36.0 (1.6)	47.6 (1.3)
**Race**			
White	10.0 (0.5)	26.2 (1.2)	33.3 (0.9)
Black	14.0 (1.0)	36.2 (4.0)	39.1 (1.8)
**Education**			
Less than high school	14.6 (1.8)	28.5 (2.5)	43.3 (2.5)
High school or vocational school	11.5 (0.9)	26.4 (2.5)	33.5 (1.2)
College and above	8.8 (0.7)	26.0 (1.9)	33.2 (1.1)
**Diabetes status**			
Normal	8.6 (0.6)	25.4 (1.8)	31.2 (1.2)
Pre-diabetes	12.4 (1.9)	23.3 (1.9)	34.8 (1.6)
Diabetes (< 10 years duration)	14.4 (2.8)	33.4 (3.9)	37.0 (1.7)
Diabetes (≥ 10 years duration)	31.5 (4.4)	41.5 (4.7)	42.3 (2.7)
**Body mass index, kg/m**^**2**^			
0–24.9	8.2 (1.0)	22.4 (1.8)	31.0 (1.4)
25–29.9	9.3 (1.0)	26.0 (1.8)	32.0 (1.2)
≥ 30	13.3 (1.1)	34.4 (2.4)	40.6 (1.4)
**Sex-specific height quartile**			
Q1	7.5 (0.9)	20.6 (1.5)	27.5 (1.5)
Q2	7.5 (0.9)	26.8 (2.1)	27.8 (1.6)
Q3	10.3 (1.0)	30.5 (2.5)	34.9 (1.6)
Q4	14.5 (1.0)	41.6 (3.5)	45.4 (1.6)
**Smoking status**			
Never	10.3 (0.8)	27.8 (1.8)	34.9 (1.2)
Former	9.8 (1.0)	27.7 (1.7)	33.8 (1.1)
Current	11.7 (1.1)	15.2 (3.4)	35.9 (3.1)
**Drinking status**			
Never	14.0 (1.9)	32.4 (3.3)	37.6 (1.9)
Former	11.7 (0.8)	28.8 (2.4)	35.4 (1.5)
Current light/moderate drinker	8.7 (0.8)	23.8 (1.7)	33.1 (1.2)
Current heavier drinker	10.8 (1.0)	21.8 (2.7)	28.8 (3.5)
**Prevalent cardiovascular disease**			
No	10.1 (0.5)	25.2 (1.6)	33.6 (0.9)
Yes	13.2 (2.2)	31.6 (2.7)	37.5 (1.8)
**Hypertension**			
No	9.4 (0.6)	26.0 (1.6)	30.3 (2.0)
Yes	11.8 (0.9)	27.2 (1.3)	35.2 (0.9)
**Hypercholesterolemia**			
No	10.1 (0.6)	29.3 (1.6)	34.2 (1.3)
Yes	11.1 (1.1)	23.6 (1.4)	34.6 (1.0)
**Prevalent chronic kidney disease**			
No	9.7 (0.5)	25.3 (1.9)	31.7 (1.0)
Yes	15.2 (1.7)	28.3 (1.9)	38.0 (1.3)
**Peripheral artery disease**^a^			
No	9.5 (0.5)	22.7 (1.4)	33.6 (0.9)
Yes	17.1 (2.8)	22.6 (2.7)	44.3 (3.8)
**Cancer**^b^			
No	10.2 (0.5)	26.0 (1.5)	34.0 (0.9)
Yes	12.0 (2.0)	29.3 (2.2)	38.0 (2.1)

^a^Prevalences are among participants who were not missing data for peripheral artery disease: N = 3215 in NHANES aged 40–69 years, N = 1204 in NHANES aged ≥ 70 years, and N = 2971 in ARIC visit 6.

^b^Prevalence for the ARIC study is among participants who were not missing cancer status in ARIC visit 6, N = 3136.

**Figure 2:**
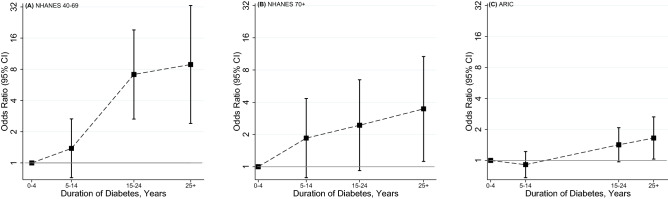
Association of peripheral neuropathy with duration of diabetes among US adults aged 40–69 (panel **A**) and ≥ 70 years (panel **B**) (NHANES, 1999–2004) and ARIC participants aged ≥ 70 years (panel **C**) (Visit 6, 2016–2017). Adjusted associations for duration of diabetes with peripheral neuropathy in ARIC (2016–2017) and NHANES (1999–2004).Odds ratios were from logistic regression models adjusted for age, sex and race. Duration of diabetes was modelled as a categorical variable: 0–4 years, 5–14 years, 15–24 years and 25 years and more. Odds ratios and 95% confidence intervals were plotted at the median of each diabetes duration category.

In both populations, PN was more common in adults with diabetes, especially among those with long-standing disease (Fig. 2). In general, risk factors for PN were similar in adults 40–69 compared to older adults and between older US adults and ARIC participants (Table 3). Age, sex, and race were strongly associated with PN, with males and blacks having a substantially higher prevalence compared to females and whites in both studies. Diabetes was also an important risk factor. The association of diabetes with PN was highest for middle-aged adults and for adults with diabetes ≥ 10 years duration, although the association of diabetes < 10 years duration with PN was also significant for all groups. Other key risk factors for PN included higher body mass index, greater height, lower education, and peripheral artery disease (in middle-aged US adults and older adults in ARIC). Never drinkers were more likely to have PN compared to current light/moderate drinkers. Prevalent cardiovascular disease, hypertension, and chronic kidney disease were moderately associated with PN.

**Table 3:** Age-, sex-, and race-adjusted odds ratios [OR (95% CI)] for the association of risk factors with peripheral neuropathy in US adults aged 40–69 and ≥ 70 years (NHANES, 1999–2004) and ARIC participants aged ≥ 70 years (Visit 6, 2016–2017).

	Aged 40–69 years	Aged ≥ 70 years	
	US adults (NHANES) OR (95% CI)	US adults (NHANES) OR (95% CI)	ARIC (Visit 6) OR (95% CI)
**Age in years**			
40–49	1 (Ref)	–	–
50–59	1.81 (1.38–2.37)	–	–
60–69	3.20 (2.38–4.31)	–	–
70–74	–	1 (Ref)	1 (Ref)
75–79	–	1.47 (1.06–2.04)	1.35 (1.06–1.71)
≥ 80	–	2.04 (1.51–2.75)	2.38 (1.87–3.02)
**Sex**			
Female	1 (Ref)	1 (Ref)	1 (Ref)
Male	2.36 (1.94–2.86)	2.22 (1.79–2.75)	2.75 (2.37–3.20)
**Race**			
White	1 (Ref)	1 (Ref)	1 (Ref)
Black	1.49 (1.20–1.85)	1.63 (1.11–2.39)	1.32 (1.09–1.58)
**Education**			
College and above	1 (Ref)	1 (Ref)	1 (Ref)
High school or vocational school	1.35 (1.01–1.81)	1.02 (0.71–1.46)	1.02 (0.87–1.19)
Less than high school	1.81 (1.26–2.58)	1.14 (0.78–1.67)	1.60 (1.25–2.03)
**Diabetes status**			
Normal	1 (Ref)	1 (Ref)	1 (Ref)
Pre-diabetes	1.53 (1.01–2.30)	0.89 (0.64–1.22)	1.19 (0.99–1.44)
Diabetes (< 10 years duration)	1.82 (1.07–3.10)	1.50 (1.00–2.27)	1.32 (1.09–1.60)
Diabetes (≥ 10 years duration)	5.26 (3.27–8.46)	2.17 (1.33–3.54)	1.68 (1.29–2.20)
**Body mass index, kg/m**^**2**^			
0–24.9	1 (Ref)	1 (Ref)	1 (Ref)
25–29.9	1.14 (0.77–1.69)	1.22 (0.93–1.61)	1.05 (0.87–1.27)
≥ 30	1.74 (1.23–2.48)	1.87 (1.39–2.51)	1.58 (1.30–1.92)
**Sex-specific height quartile**			
Q1	1 (Ref)	1 (Ref)	1 (Ref)
Q2	0.99 (0.68–1.44)	1.43 (1.11–1.85)	1.02 (0.81–1.28)
Q3	1.43 (1.05–1.93)	1.74 (1.27–2.39)	1.46 (1.17–1.81)
Q4	2.15 (1.54–3.00)	2.91 (2.04–4.14)	2.36 (1.91–2.91)
**Smoking status**			
Never	1 (Ref)	1 (Ref)	1 (Ref)
Former	0.95 (0.70–1.30)	0.99 (0.74–1.31)	0.95 (0.81–1.11)
Current	1.17 (0.86–1.59)	0.45 (0.26–0.80)	1.05 (0.77–1.42)
**Drinking status**			
Current light/moderate drinker	1 (Ref)	1 (Ref)	1 (Ref)
Current heavier drinker	1.28 (0.93–1.76)	0.89 (0.60–1.32)	0.80 (0.55–1.17)
Former	1.41 (1.07–1.84)	1.31 (0.93–1.84)	1.11 (0.93–1.33)
Never	1.74 (1.21–2.49)	1.56 (1.06–2.30)	1.24 (1.01–1.52)
**Prevalent cardiovascular disease**			
No	1 (Ref)	1 (Ref)	1 (Ref)
Yes	1.37 (0.90–2.08)	1.39 (0.96–2.00)	1.20 (1.00–1.44)
**Hypertension**			
No	1 (Ref)	1 (Ref)	1 (Ref)
Yes	1.30 (1.02–1.67)	1.07 (0.86–1.32)	1.27 (1.03–1.58)
**Hypercholesterolemia**			
No	1 (Ref)	1 (Ref)	1 (Ref)
Yes	1.12 (0.84–1.48)	0.74 (0.59–0.92)	1.02 (0.88–1.19)
**Prevalent chronic kidney disease**			
No	1 (Ref)	1 (Ref)	1 (Ref)
Yes	1.71 (1.25–2.33)	1.17 (0.84–1.62)	1.35 (1.16–1.57)
**Peripheral artery disease**^a^			
No	1 (Ref)	1 (Ref)	1 (Ref)
Yes	2.02 (1.31–3.10)	0.99 (0.69–1.42)	1.64 (1.16–2.30)
**Any cancer**^b^			
No	1 (Ref)	1 (Ref)	1 (Ref)
Yes	1.21 (0.79–1.84)	1.19 (0.88–1.59)	1.21 (0.98–1.48)

^a^Odds ratios are among participants who were not missing data for peripheral artery disease: N = 3215 in NHANES aged 40–69 years, N = 1204 in NHANES aged ≥ 70 years, and N = 2971 in ARIC visit 6.

^b^Odds ratio for ARIC is among participants who were not missing cancer status in ARIC visit 6, N = 3136.

Simultaneous adjustment for all cardiovascular and diabetes factors did not substantially alter our findings, with a few exceptions (Supplemental Table 1). First, the associations of black race and hypertension with PN were no longer significant after adjustment for other risk factors. Second, only long-standing diabetes (≥ 10 years duration) remained associated with PN after multivariable adjustment.

## Discussion

Based on data from NHANES and ARIC, we found that the age, sex, and race-adjusted prevalence of PN defined by monofilament sensitivity for adults aged 70 years or older was 26.8% in US adults and 34.4% in the ARIC Study compared to 10.4% in US adults aged 40–69 years. Risk factors associated with PN in both NHANES and ARIC included long-standing diabetes, older age, male sex, black race, higher body mass index, and greater height. There were weaker associations of hypertension, prevalent cardiovascular disease, prevalent chronic kidney disease, and peripheral artery disease, particularly among middle-aged US adults and older ARIC participants. Notably, although diabetes was significantly associated with PN for all groups, PN was also present in a large proportion of adults without diabetes in both the US population and ARIC. This finding suggests that PN defined by monofilament sensitivity may affect a greater proportion of adults without diabetes than previously appreciated.

The higher prevalence of PN among older ARIC participants as compared to NHANES may be due to several factors. First, the ARIC and NHANES populations are different. Participants in ARIC had more comorbidities than in NHANES, potentially due to differences in study recruitment and selection. Second, a higher proportion of older ARIC versus NHANES participants had diabetes, the most commonly described risk factor for PN.

We observed a strong age-related increase in the prevalence of PN. There are a several studies documenting an association of age with PN among adults with diabetes^5,22^. Although other reports on the epidemiology of PN in older adults in the general population are scarce, there are a several studies documenting strong associations of age with PN among adults with diabetes. Among 6487 adults with diabetes in the United Kingdom, the prevalence of PN was approximately 5% in the 20–29 year age group, compared to 44% in the 70–79 year age group^5^. Cabezas-Cerrato performed a cross-sectional study of PN among 2644 adults with diabetes in Spain, and reported that 30% of those aged 70–74 years had PN^22^. These estimates are similar to the prevalence estimates that we report among older adults in NHANES and ARIC, although the variability among studies likely reflects heterogeneity in recruitment approaches, the populations studied, and diagnostic protocols for PN.

Our study confirms previous findings, but also demonstrates that PN is highly prevalent in older adults without diabetes. The American Diabetes Association recommends annually assessment for PN in all adults with diabetes^18^, but PN screening with monofilament testing is not routinely performed in adults without diabetes. In a separate study, we recently demonstrated a strong and independent association of PN with mortality in both persons with and without diabetes^9^. Taken as whole, our results suggest that the high prevalence of PN is common and is an underrecognized risk factor for mortality in the general population.

Some of the risk factors with PN we identified in older adults have been previously described in younger populations, including body mass index, hypertension, history of cardiovascular disease, and peripheral artery disease^23–25^. Tesfaye et al. performed a prospective study of 1172 patients with type 1 diabetes with 7.3 years of follow-up (mean age 32.7 years), and found that the risk of PN was independently associated with duration of diabetes, glucose control, body mass index, smoking, and prevalent cardiovascular disease^23^. Olaiya et al. recently performed a cross-sectional study of middle-aged adult volunteers (mean age 41.9 years), and found that age, male sex, body mass index, heart rate, mean arterial pressure, and family history of diabetes or cardiovascular diseases were important risk factors^26^. We did not find an association of hypercholesterolemia with PN among older adults, but we did find strong differences by sex and race. There was also an inverse association between smoking and PN in older adults in NHANES. While smoking and peripheral neuropathy were positively associated in a recent meta-analysis^27^, there are prior studies that report an inverse relationship similar to ours^28,29^. This may be due to a survival bias or possibly a quitting effect, where current smoking is more common among those with better health or among surviving older patients^29^.

The prevalence of PN was high among adults with diabetes and positively associated with longer duration of diabetes. This finding is consistent with previously published PN data from the general population^2,16,17^. Prior data also show convincing evidence that a longer duration of diabetes is associated with a higher prevalence of peripheral neuropathy^23,30^, and we found a similar association of diabetes duration with PN in both middle-aged and older adults. The stronger association of PN with diabetes in middle-aged compared to older adults observed in our study is consistent with the large literature comparing the contribution of vascular risk factors in mid-life to those in old-age^31–35^. Older adults have substantially more cardiometabolic disease and risk factors compared to middle-aged adults^13^; thus, any single risk factor tends to have relatively lower effect in older as compared to younger ages. It is also possible that PN in older adults represents a microvascular etiology of disease that is less dependent on hyperglycemia than other cardiovascular risk factors. Consistent with this notion, we have previously shown strong associations of cardiac troponin T and N-terminal (NT)-pro hormone BNP with PN in older adults both with and without diabetes^36^.

Because PN is classically attributed to hyperglycemia^37^, the high prevalence of PN in non-diabetic adults in our study is novel and warrants attention. PN is a side effect of chemotherapy and can result from chronic alcoholism, viral infections, spinal cord injury, or radiculopathy as well^38–41^. However, these conditions are uncommon in the general population and are unlikely to explain the high prevalence of PN that we observed in the NHANES and ARIC study populations. We did find a slightly higher prevalence of PN among adults with a history of cancer in our study. Peripheral nerve dysfunction has been reported to occur in 25–66% of chronic alcoholics in the US^41^, but this association is unlikely to be generalizable to more moderate drinking. We did not find an association of former drinking with PN in our study; in fact, adults with no history of drinking had the highest prevalence of PN. The explanation for this finding is unclear, but may reflect differences between drinkers and non-drinkers and that moderate drinking is typically associated with beneficial cardiometabolic risk^41,42^. Regardless of the etiology, the high prevalence of non-diabetic PN among adults ≥ 70 years of age suggests that there may be an unexplained loss of peripheral sensation among older adults (i.e. idiopathic PN) that is underrecognized. Notably, there are no formal guidelines for foot examinations or monofilament testing among adults without diabetes. The vast majority of data available describing PN risk factors and outcomes are in populations of adults with diabetes^43–46^. More studies are needed to understand the underlying etiology and clinical implications of idiopathic PN in older adults.

Novel aspects of our study include the characterization of the epidemiology and risk factors associated PN among middle-aged and older adults, separately, and stratified by diabetes status. There have been a number of cohort studies that have previously reported the prevalence and risk factors associated with PN. Most of these studies have focused on either middle-aged adults or older adult men or women (separately). Our study adds to the existing literature by describing the prevalence of and risk factors associated with PN, defined by monofilament insensitivity, among older adults (> 70 years of age) both with and without diabetes. These data are important to our understanding of the epidemiology of PN, and show that the risk factors for PN in middle age are not entirely the same as the risk factors for PN in older adults.

The limitations of our study include a lack of symptom-based data in both NHANES and ARIC. Our evaluation of peripheral sensation was limited to monofilament testing only; neither NHANES or ARIC used nerve conduction studies, vibration threshold testing, or patient-reported questionnaires such as the Michigan Neuropathy Screening Instrument^47,48^ to capture symptoms. As a result, our findings are limited to an evaluation of PN with decreased lower extremity sensation only, which may be an advanced form of the disease^19^. There were also some minor differences in covariate assessments between NHANES and ARIC. Variation in covariate definitions may explain some of the differences in associations (e.g. prevalent cardiovascular disease). However, both NHANES and ARIC used trained personnel and standardized approaches to data collection, and variable definitions were similar for most conditions. Importantly, PN was evaluated using the same monofilament testing protocols in both studies.

In conclusion, PN defined by monofilament insensitivity is highly prevalent in the general population, especially among older adults. Long-standing diabetes, male sex, and black race are strong risk factors for PN in both middle-aged and older adults in the general population. Older age is also closely associated with PN in adults with and without diabetes, the latter of which has previously been unrecognized. Future studies should evaluate prognosis in older adults with PN in the absence of diabetes, and determine whether screening for PN using monofilament testing in older adults, regardless of diabetes status, affects important clinical outcomes.

## Supplementary Information

Supplementary Table 1.
