# Supplementary material for: Prevalence of peripheral neuropathy defined by monofilament insensitivity in middle-aged and older adults in two US cohorts
Source: Sci Rep. 2021 Sep 27;11:19159. doi: 10.1038/s41598-021-98565-w (PMC8476511; doi:10.1038/s41598-021-98565-w)
Supplement: Supplementary file 1 — Supplementary Table 1. [file 41598_2021_98565_MOESM1_ESM.docx]

**Supplementary Table 1. Multivariable^a^ Logistic Regression for Association [OR (95% CI)] of Peripheral Neuropathy and Potential Risk Factors in U.S. Adults Aged 40-69 and ≥70 Years (NHANES, 1999-2004) and ARIC Participants Aged ≥70 Years (Visit 6, 2016-2017)**

|  | **Aged 40-69 Years** | **Aged ≥70 Years** | |
| --- | --- | --- | --- |
|  | **U.S. Adults**  **(NHANES)**  **OR (95% CI)** | **U.S. Adults**  **(NHANES)**  **OR (95% CI)** | **ARIC**  **(Visit 6)**  **OR (95% CI)** |
| Age in years |  |  |  |
| 40-49 | 1 (ref) | -- | -- |
| 50-59 | 1.74 (1.28-2.36) | -- | -- |
| 60-69 | 2.68 (1.89-3.81) | -- | -- |
| 70-74 | -- | 1 (ref) | 1 (ref) |
| 75-79 | -- | 1.52 (1.09-2.12) | 1.38 (1.07-1.78) |
| ≥80 | -- | 2.53 (1.84-3.47) | 2.45 (1.90-3.17) |
| Sex |  |  |  |
| Female | 1 (ref) | 1 (ref) | 1 (ref) |
| Male | 2.52 (1.99-3.20) | 2.34 (1.76-3.11) | 2.87 (2.43-3.38) |
| Race |  |  |  |
| White | 1 (ref) | 1 (ref) | 1 (ref) |
| Black | 1.07 (0.80-1.41) | 1.44 (0.94-2.22) | 1.00 (0.81-1.22) |
| Education |  |  |  |
| College and above | 1 (ref) | 1 (ref) | 1 (ref) |
| High school or vocational school | 1.40 (1.03-1.90) | 1.04 (0.73-1.49) | 1.02 (0.86-1.21) |
| Less than high school | 1.84 (1.25-2.71) | 1.10 (0.76-1.60) | 1.66 (1.28-2.15) |
| Diabetes status |  |  |  |
| Normal | 1 (ref) | 1 (ref) | 1 (ref) |
| Pre-diabetes | 1.42 (0.93-2.16) | 0.87 (0.61-1.25) | 1.18 (0.97-1.45) |
| Diabetes (<10 years duration) | 1.56 (0.93-2.62) | 1.27 (0.84-1.91) | 1.22 (0.99-1.50) |
| Diabetes (≥10 years duration) | 4.50 (2.69-7.51) | 1.86 (1.18-2.91) | 1.52 (1.14-2.02) |
| Body mass index, kg/m^2^ |  |  |  |
| 0-24.9 | 1 (ref) | 1 (ref) | 1 (ref) |
| 25-29.9 | 1.13 (0.76-1.69) | 1.21 (0.93-1.58) | 1.02 (0.84-1.24) |
| ≥30 | 1.47 (1.01-2.15) | 1.83 (1.31-2.54) | 1.47 (1.20-1.81) |
| Sex-specific height quartile |  |  |  |
| Q1 | 1 (ref) | 1 (ref) | 1 (ref) |
| Q2 | 1.07 (0.73-1.59) | 1.51 (1.17-1.95) | 1.08 (0.85-1.37) |
| Q3 | 1.77 (1.29-2.42) | 1.84 (1.33-2.54) | 1.59 (1.27-2.00) |
| Q4 | 2.83 (2.04-3.92) | 3.24 (2.29-4.57) | 2.58 (2.07-3.22) |
| Smoking status |  |  |  |
| Never | 1 (ref) | 1 (ref) | 1 (ref) |
| Former | 0.92 (0.66-1.28) | 1.04 (0.72-1.49) | 0.97 (0.82-1.15) |
| Current | 1.11 (0.79-1.57) | 0.51 (0.28-0.92) | 1.10 (0.79-1.52) |
| Drinking status |  |  |  |
| Current light/moderate drinker | 1 (ref) | 1 (ref) | 1 (ref) |
| Current heavier drinker | 1.20 (0.87-1.66) | 0.93 (0.63-1.38) | 0.86 (0.58-1.27) |
| Former | 1.10 (0.80-1.52) | 1.30 (0.93-1.81) | 1.04 (0.86-1.26) |
| Never | 1.48 (1.01-2.15) | 1.51 (0.99-2.30) | 1.17 (0.93-1.46) |
| Prevalent cardiovascular disease |  |  |  |
| No | 1 (ref) | 1 (ref) | 1 (ref) |
| Yes | 1.05 (0.71-1.55) | 1.40 (0.99-2.00) | 1.12 (0.93-1.37) |
| Hypertension |  |  |  |
| No | 1 (ref) | 1 (ref) | 1 (ref) |
| Yes | 1.07 (0.82-1.40) | 0.99 (0.78-1.26) | 1.15 (0.91-1.45) |
| Hypercholesterolemia |  |  |  |
| No | 1 (ref) | 1 (ref) | 1 (ref) |
| Yes | 1.01 (0.75-1.34) | 0.70 (0.55-0.89) | 0.90 (0.77-1.07) |
| Prevalent chronic kidney disease |  |  |  |
| No | 1 (ref) | 1 (ref) | 1 (ref) |
| Yes | 1.29 (0.93-1.78) | 1.05 (0.77-1.44) | 1.23 (1.05-1.45) |

*^a^ Mutually adjusted for all variables in the table*
